# Extensive myocardial infarction complicated with stroke as the first presentation of HIV in A young sudanese male: A case report

**DOI:** 10.1016/j.amsu.2022.104653

**Published:** 2022-09-15

**Authors:** Mohammed Mahmmoud Fadelallah Eljack, Najla Fouad Nassir Mohammedali, Kabab Abbasher Hussien Mohamed Ahmed, Omer Idris Ahmed, Alshareef B. Nour, Mazin S. Haroun, Abdallah M. Abdallah, Mustafa Mohamed Ibrahim Ali

**Affiliations:** a University of Bakht Alruda, Ad Duwaym, Sudan; b Medani Heart Center, Sudan; c Univeristy of Khartoum, Sudan; d University of Alfashir, Medical Doctor at Medani Heart Center, Sudan; e Wad Madani College of Medical Science and Technology, Sudan; f University of Khartoum, Sudan; g University of Bahri, Sudan

**Keywords:** HIV, Myocardial infarction, Stroke, Sudan

## Abstract

**Introduction:**

As it is a disseminated disease, HIV infection can be associated with significant cardiovascular and neurological complications; however, this commonly occurs late. Here, we highlight the unusual initial presentation of HIV infection, which is myocardial infarction complicated by stroke.

**Case presentation:**

A 30 years old male with a clear medical background presented with severe chest pain with evidence of ischemia on ECG and positive serum troponin. he received anti-ischemic drugs, and was prepared for coronary angiography with routine investigations tested positive for HIV; however, his condition was later complicated by stroke.

**Discussion:**

Antiretroviral medication, HIV disease characteristics, female gender, and HCV co-infection are risk factors for coronary artery disease (CAD) in HIV patients. Patients living with HIV are also at risk of developing stroke, which can be caused by atherosclerosis of the major arteries, small artery disease, cardiac embolism, CNS infections, coagulation issues, and non-atherosclerotic vasculopathy.

**Conclusion:**

The presentation of an acute coronary syndrome in a young patient should raise suspicion of uncommon causes and needs a prompt evaluation from digging up in history, detailed examination, and investigations with close follow-up to prevent the complications that may occur. on the other hand, known HIV Patients should be screened periodically with an electrocardiogram.

## Highlights

• HIV infection can be associated with significant cardiovascular and neurological complications.
• The presentation of acute coronary syndrome in a young patient should raise suspicion of uncommon causes.
• Known HIV Patients should be screened periodically with electrocardiogram.

## 1 Introduction

Patients with HIV are more likely to develop coronary artery disease (CAD) owing to classic CAD risk factors, antiretroviral medication effects, or HIV-related characteristics such as inflammatory and immunological alterations [1]. In addition, HIV-related intra- and extracranial vasculopathy, HIV-induced cardiomyopathy, HIV-induced coagulopathy, and opportunistic infection-associated vasculitis have been linked to an increased risk of stroke; however, this is more prevalent in people under the age of 50 years, but not in those aged 50 years [2,3]. Slim illness (diarrhea and wasting), Tuberculosis, a variety of opportunistic infections (OI), weight loss, fever, and dermatological symptoms are among the most common early manifestations of HIV infection in Africans [4]. We present the case of a young Sudanese man who was diagnosed with Extensive ST-segment Elevation Myocardial Infarction (STEMI) later worsened by Ischemic Stroke and was found to have a high HIV viral load after ruling out all possible explanations of his underlying disease. This case report is compliant with the SCARE Guidelines 2020 [5].

## 2 Case report

A 30-year-old thin male with a clean medical history was referred by ambulance from Wad Medani Teaching Hospital to the Medani Heart Center (MHC) emergency department with central crushing chest pain that was aggravated by movement and improved by rest. The pain was not associated with cough or shortness of breath, and it was not improved by food or Antacids. Fever, change in appetite, diarrhea, vomiting, change in urine, headache, or any other neurological symptoms were denied by the patient.

There had been no prior history of a similar disease, cardiac disease, psychiatric problem, or surgical procedure. There is no history of a similar condition or genetic abnormality in the family. The patient stated that he is a heavy smoker who has had numerous instances of unprotected sexual intercourse. The patient stated that he did not consume alcohol. The patient was not on short-term or long-term medications.

The patient appeared unwell, skinny, and not pale or jaundiced, with a pulse of 110 regular good volume, Bp of 100/60 bilaterally, normal pericardium findings, a clear chest, and a soft abdomen. The ECG revealed significant ST segment elevation from v1 to v6, as well as mild elevation in leads 2, 3, and avF, which was later confirmed by a positive serum troponin as Late Extensive ST-segment elevation. Myocardial infarction was crushed by aspirin 300 mg, clopidogrel 600 mg, bisoprolol 2,5 mg, and Enoxaparin injection 0.5mg/kg 12 hourly, and the patient was scheduled for urgent coronary angiography by the attending senior cardiologist, but during preparation rapid immunochromatographic test for detection of antibodies to Human Immunodeficiency Virus (ICT) tested positive, necessitating confirmation with ELISA, which revealed a high viral load of 22.4 AU/ml (normal up to 1 AU/m). Other tests revealed a normal fasting lipid profile, complete blood count, and electrolyte-based kidney function tests.

**Fig. 1:**
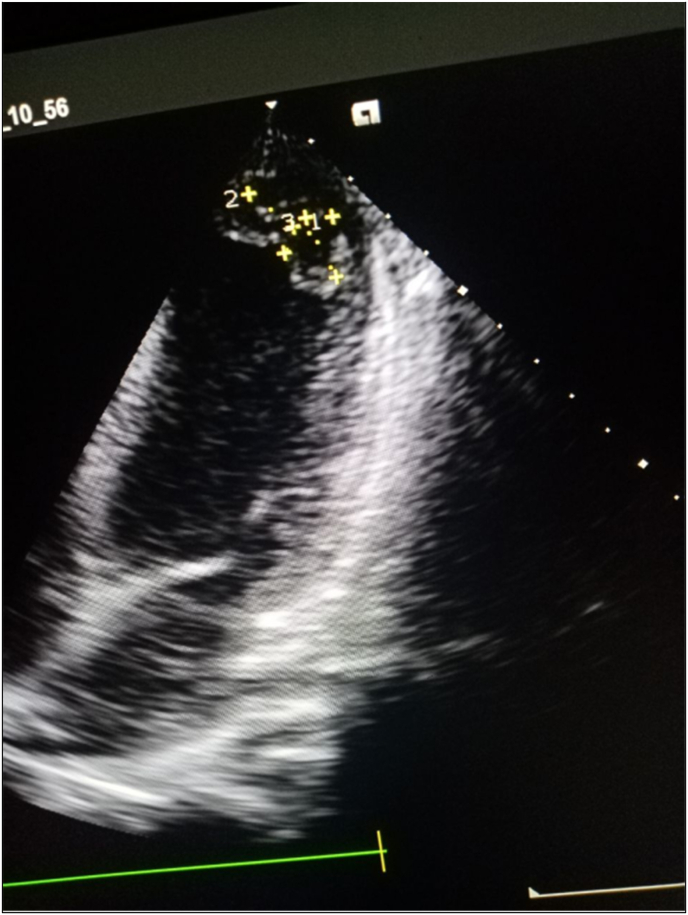
Echocardiography showed anterior, septal, apical and anterolateral wall hypokinesia.

With moderately impaired left ventricular systolic function (Ejection Fraction = 34) and anterior, septal, apical, and anterolateral wall hypokinesia, as well as two apical thrombus measures 11 × 10 cm and 10 × 12 cm subsequently, and moderately impaired left ventricular systolic function (Ejection Fraction = 34), the decision to add furosemide 40 mg tablets once daily and spironolactone tablets 25 mg. See Fig. 1.

**Fig. 2:**
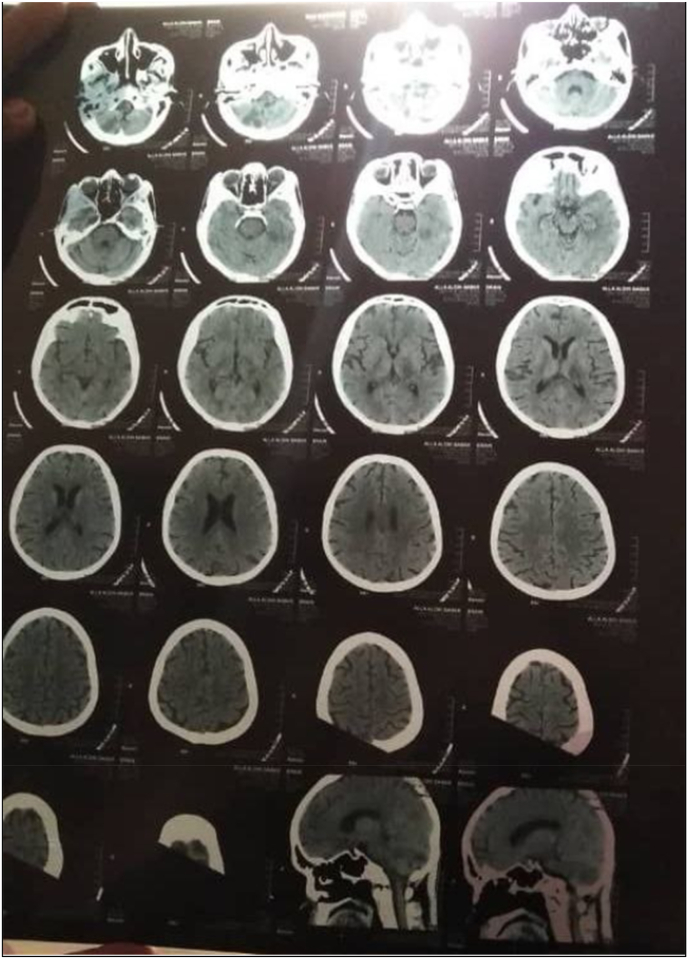
Brain CT Scan which showed left parietal area infarction.

The patient was closely monitored symptomatically with serial ECGs on subsequent days, which showed significant improvement until the evening follow-up on day 4 of admission when he was found to have slurred speech and was unable to move his right side without mouth deviation, headache, or blurring of vision. He was afebrile, conscious, and oriented in time, place, and person during the evaluation. Bulbar cranial nerve affection (without facial palsy), hypotonia on the right side, power grade 3, and hyporeflexia were all present. The patient was taken for an emergency brain CT scan, which revealed an infarction in the left parietal area. See Fig. 2.

The patient had no previous CT brain since he had never experienced any symptoms or felt the need to do one. Carotid angiography was not performed due to a scarcity of resources. The changes in the CT brain were related to the patient's stroke symptoms. The patient was observed for the next five days and showed total recovery, with normal tone, power grade 5, and reflexes. On day 10 of admission, he was discharged after being properly educated on comprehensive anti-ischemic and diuretic therapy with enoxaparin switched to rivaroxaban 15 mg 12 hourly, and assigned to the HIV program for proper management, to be examined in the Cardiology Referral Clinic after two weeks.

## 3 Discussion

Patients with HIV are more likely to develop coronary artery disease (CAD), although the short-term prognosis following a heart attack or stroke is unknown [6]. Antiretroviral medication, HIV disease characteristics, female gender, and HCV co-infection were all described as HIV-related coronary heart disease risk factors in the literature [7]. Inflammation, endothelial dysfunction, and coagulation abnormalities all have a role in the development of atherosclerosis in HIV-positive patients [8]. The intensity of HIV-related immunosuppression is linked to several anomalies that explain the observed hypercoagulability in HIV-positive patients. CD4^+^ cell counts and the presence of infectious or neoplastic disorders are used to determine this relationship. Natural anticoagulants/heparin cofactor dimer levels are lower, antiphospholipid antibodies/lupus anticoagulant activity is higher, factor VIII coagulant activity is higher, activated protein C resistance is higher, platelet activation is higher, and hyperhomocysteinemia is higher [9,10]. To our knowledge, This is the first case to be reported regarding this topic worldwide. The ECG in our patient exhibited significant ST segment elevation from v1 to v6, as well as modest elevation in leads 2, 3, and avF, which was confirmed by a positive serum troponin as Late Extensive ST-segment elevation Myocardial infarction. Although myocardial infarction was our primary working hypothesis, we considered a wide range of other possibilities. We initially ruled out HIV opportunistic infections because myocardial infarction in HIV patients is typically seen in the elderly, aged 50 and more, according to research, as opposed to our 30-year-old previously healthy patient [8]. However, during a normal outpatient HIV testing program in the emergency room before the urgent coronary angiography, the ICT result for HIV was positive, and this was confirmed by an ELISA test that revealed a high viral load of 22.4 AU/ml [[11], [12], [13]]. He had a stroke after his myocardial infarction, mostly due to embolism of the mural thrombus seen in his Echo, but he was treated and stabilized. HIV is linked to other risk factors for strokes, such as immunosuppression and high viremia, which our patient had. The etiology of stroke in HIV-positive persons is multifaceted, including atherosclerosis of the major arteries, small artery disease, cardiac embolism, CNS infections, coagulation problems, and non-atherosclerotic vasculopathy, however, the triggering mechanism for stroke remains unknown [14]. The myocardial infarction was directly associated with HIV chronic inflammation and immunological activation, independent of other risk variables, according to one possible explanation for the presentation at this age [7]. Two cohort studies demonstrated a clear link between HIV and an elevated risk of myocardial infarction of 44%–48%, regardless of established risk variables such as age, race, socioeconomic position, and substance misuse [15,16]. This is the first example of atypical HIV presentation as an ST-elevation Myocardial Infarction complicated by a stroke at such a young age that we are aware of.

## 4 Conclusion

This is a rare and unique presentation of a young HIV-positive patient. It emphasizes the possibility of subclinical HIV infection, as well as the elevated risk of coronary artery disease and stroke in this HIV-positive population of young people. It also emphasizes the importance of routine out-patient HIV testing in such emergencies, as it reduces the chances of debilitating diseases going undiagnosed, only to be discovered by severe complications at a very late stage, as in the case of a patient diagnosed with HIV for the first time at the age of 74 [17]. Screening tests for unusual presentations prevent fatal complications of common diseases.

## Data availability statement

The data that support the findings of this paper is available with the corresponding author upon reasonable request.

## Publication history

Authorea Preprint's Link: https://www.authorea.com/users/439069/articles/548427-extensive-myocardial-infarction-complicated-with-stroke-as-first-presentation-of-hiv-in-a-young-sudanese-male-case-report.

## Provenance and peer review

Not commissioned, externally peer-reviewed.

## Ethical approval

Ethical approval was obtained from the research and ethics committee at Medani Heart Center (MHC).

## Please state any sources of funding for your research

The study was self funded.

## Author contribution

MMF, NFN, OIA and ABN: Took full detailed history, did Examinations and Investigations, KAH, MSH, AMA and MMI: Wrote first draft and finalized the draft, All authors revised the final draft and contributed significantly to this study.

## Please state any conflicts of interest

Authors report no conflict of interest.

## Registration of research studies

1. Name of the registry: Unique Identifying number or registration ID: Hyperlink to your specific registration (must be publicly accessible and will be checked).

## Guarantor

Mohammed Mahmmoud Fadelallah Mohammed.

University of Bakht Alruda, Faculty of Medicine, Ad Duwaym, Sudan.

Wad medani, Gezira State, Postal code: 11112, Sudan, Mobile: 00249964656914,

m.mahmmoud96@gmail.com,

ORCID: 0000-0002-2370-9368.

## Consent

Written informed consent was obtained from the patient for publication of this case report and any accompanying images. A copy of the written consent is available for review by the editor-in-chief of this journal.

## List of Abbreviations

HIV: Human Immunodeficiency Virus
ELISA: enzyme-linked immunosorbent assay
ICT: immunochromatography test
